# A Comprehensive Association Analysis of Homocysteine Metabolic Pathway Genes in Singaporean Chinese with Ischemic Stroke

**DOI:** 10.1371/journal.pone.0024757

**Published:** 2011-09-15

**Authors:** Hui-Qi Low, Christopher P. L. H. Chen, Katherine Kasiman, Anbupalam Thalamuthu, Seok-Shin Ng, Jia-Nee Foo, Hui-Meng Chang, Meng-Cheong Wong, E-Shyong Tai, Jianjun Liu

**Affiliations:** 1 Human Genetics, Genome Institute of Singapore, Singapore; 2 Department of Pharmacology, National University of Singapore, Singapore; 3 Centre for Molecular Epidemiology, National University of Singapore, Singapore; 4 Department of Medical Epidemiology and Biostatistics, Karolinska Institutet, Stockholm, Sweden; 5 Department of Neurology, National Neuroscience Institute, Singapore; 6 Division of Medical Sciences, National Cancer Centre, Singapore; Ohio State University Medical Center, United States of America

## Abstract

**Background:**

The effect of genetic factors, apart from 5,10-methylenetetrahydrofolate reductase (*MTHFR*) polymorphisms, on elevated plasma homocysteine levels and increasing ischemic stroke risk have not been fully elucidated. We conducted a comprehensive analysis of 25 genes involved in homocysteine metabolism to investigate association of common variants within these genes with ischemic stroke risk.

**Methodology/Principal Findings:**

The study was done in two stages. In the initial study, SNP and haplotype-based association analyses were performed using 147 tagging Single Nucleotide Polymorphisms (SNPs) in 360 stroke patients and 354 non-stroke controls of Singaporean Chinese ethnicity. Joint association analysis of significant SNPs was then performed to assess the cumulative effect of these variants on ischemic stroke risk. In the replication study, 8 SNPs were selected for validation in an independent set of 420 matched case-control pairs of Singaporean Chinese ethnicity. SNP analysis from the initial study suggested 3 risk variants in the *MTRR*, *SHMT1* and *TCN2* genes which were moderately associated with ischemic stroke risk, independent of known stroke risk factors. Although the replication study failed to support single-SNP associations observed in the initial study, joint association analysis of the 3 variants in combined initial and replication samples revealed a trend of elevated risk with an increased number of risk alleles (Joint *P*_trend_ = 1.2×10^−6^).

**Conclusions:**

Our study did not find direct evidence of associations between any single polymorphisms of homocysteine metabolic pathway genes and ischemic stroke, but suggests that the cumulative effect of several small to moderate risk variants from genes involved in homocysteine metabolism may jointly confer a significant impact on ischemic stroke risk.

## Introduction

Ischemic stroke is a complex multifactorial disease influenced by several genetic and environmental factors [1]–[2]. Epidemiological studies have identified several clinical risk factors that are highly correlated with stroke risk; these in turn implicate various biological pathways, including the homocysteine metabolism[3], lipid metabolism[4], renin-angiotensin-aldosterone system[5]–[6], haemostatic system[6], phosphodiesterase 4D[7] and leukotriene pathways[8], in stroke pathophysiology. Moderate elevation of plasma homocysteine levels has been identified as a major risk factor for vascular diseases, including stroke[3]. Data from cohort and case-control studies suggest a positive, and dose-dependent association between the serum concentration of total homocysteine and the risk of stroke, which is independent of other vascular risk factors[9]. In meta-analyses of large cohorts, it has been estimated that for every rise in plasma homocysteine level of 5 umol/L, the risk for cerebrovascular disease increases by 50% (95% CI, 30- 90%) while plasma homocysteine levels above the 95^th^ percentile was associated with an odds ratio (OR) of 3.97 (95% CI, 3.07 to 5.12) for cerebrovascular disease[10]–[11].

Mutations in genes of the homocysteine metabolic pathway may confer an increased risk for ischemic stroke as a consequence of elevated plasma homocysteine levels. A common polymorphism (C677T) in the gene encoding 5,10-methylenetetrahydrofolate reductase (*MTHFR*), a critical enzyme in homocysteine metabolism, has been reported by several studies to be associated with both elevated plasma homocysteine levels and increased stroke risk[12]–[13]. A recent meta-analysis further confirmed the association with variation in plasma homocysteine levels (weighted mean difference in homocysteine levels of 1.93 µmol/L for TT vs CC) and increased risk of stroke (OR 1.26 for TT vs CC)[14]. However, the C677T polymorphism alone accounts for less than 10% of the variance in plasma homocysteine levels[3].

Several polymorphisms in other homocysteine regulatory genes have also been investigated, but their effects on plasma homocysteine remain unclear. The A2756G polymorphism in 5-methyltetrahydrofolate-homocysteine methyltransferase (*MTR*) was identified to be associated with decreased plasma homocysteine levels, but only in some populations[15]–[16]. The first genome-wide linkage analysis of plasma homocysteine levels, performed in a Spanish population, found that the nicotinamide N-methyltransferase (*NNMT*) gene was the most significant genetic determinant of plasma homocysteine[17]. A second genome wide linkage study, performed in non-Hispanic whites and blacks, identified several genomic regions (non-Hispanic whites: chromosomes 6q26 and 18q21, blacks: chromosome 2q32, 7p15, 8q24, 18q21, and 20p12) showing linkage with plasma homocysteine levels[18]. However, the results from these two genome-wide linkage studies are largely inconsistent. Furthermore, the roles of these polymorphisms and linkage loci in stroke risk have not been evaluated.

Postulating that there are additional genetic risk variants other than the *MTHFR* C677T variant that elevate plasma homocysteine levels and thus increase risk for ischemic stroke, we performed a comprehensive genetic association study of the homocysteine metabolic pathway by investigating 25 homocysteine metabolic pathway genes in well-characterized ischemic stroke cases and matched controls of Singaporean Chinese ethnicity. This is the first comprehensive study to be performed in a Chinese population, which has been underrepresented in the earlier linkage and association studies.

## Results

### Initial Study

We analyzed 147 tagging SNPs using the Cochrane-Armitage trend tests to obtain ORs and 95% Confidence Intervals (CI) (*Table S1*). After an adjustment for other known non-genetic risk factors (vascular risk factors, age and gender), rs16879248 in *MTRR* (5-methyltetra-hydrofolate-homocysteine methyltransferase reductase), rs11868708 in *SHMT1* (serine hydroxymethyl-transferase 1) and rs11703570 in *TCN2* (transcobalamin II), show suggestive association with ischemic stroke risk (*P*<0.05) (*Table 2*). While rs16879248 showed a protective effect (OR = 0.67), rs11868708 and rs11703570 were associated with increased risk for ischemic stroke (OR = 1.46 and 1.51 respectively). The associations at all 3 SNPs appear to fit an additive or dominant model (*Table S4*).

**Table 1:** Baseline Characteristics of Control Subjects and Ischemic Stroke Patients.

	Candidate Gene Study			Replication Study		
Characteristics	Control(n = 354)	Ischemic Stroke(n = 360)	*P*	Control(n = 420)	Ischemic Stroke(n = 420)	*P*
Gender, male, n(%)	161(45.5%)	232(64.4%)	<0.0001	284(67.6%)	292(69.5%)	0.5520
Smokers, n(%)	67(19.0%)	104(28.9%)	0.0021	112(26.7%)	163(38.8%)	<0.0001
Hypertension, n(%)	174(49.3%)	264(73.3%)	<0.0001	194(46.2%)	306(72.9%)	<0.0001
Diabetes, n(%)	72(20.3%)	117(32.5%)	<0.0001	13(3.1%)	172(41.0%)	<0.0001
Hyperlipidemia, n(%)	159(45.4%)	170(47.2%)	0.5103	106(25.2%)	249(59.3%)	<0.0001
Mean Age, years ±SD	59.5±9.9	64.4±11.7	<0.0001	53.6±9.2	55.73±9.3	0.0013

In addition to single SNP-based association analysis, we performed haplotype analysis of 19 candidate genes. As shown in *Table 3*, results from the haplotype analysis were largely consistent with the single SNP analysis for *SHMT1* and *TCN2*. We also analyzed pair-wise interaction among the 3 associated SNPs as well as all the 147 tagging SNPs, but no evidence of interaction was found.

**Table 2:** Characteristics of Significant SNPs and Their Association with Ischemic Stroke Risk under Additive Model.

Initial Study						Replication Study				Combined Analysis		
dbSNP ID	Gene	Alleles^a^	MAF^b^	P	OR^c^	TagSNP	MAF^b^	P	OR^c^	MAF^b^	P	OR^c^
					(95% CI)				(95% CI)			(95% CI)
rs16879248	*MTRR*	T/C	0.25:0.19	0.01	0.67(0.50-0.89)	rs16879259^d^	0.19:0.17	0.29	0.85(0.63-1.15)	0.22:0.18	0.004	0.75(0.61-0.91)
rs11868708	*SHMT1*	T/C	0.30:0.36	0	1.46(1.15-1.86)	rs9909104^e^	0.31:0.34	0.62	1.06(0.84-1.35)	0.31:0.35	0.01	1.24(1.05-1.46)
rs11703570	*TCN2*	T/A	0.18:0.22	0.01	1.51(1.12-2.02)	rs2301955^f^	0.18:0.19	0.36	1.15(0.85-1.56)	0.18:0.20	0.018	1.28(1.04-1.57)

a Major alleles given first, minor alleles second.

b Case:Control.

c Adjusted risk factors: gender, age, hypertension, diabetes, hyperlipidemia, smoking.

d perfectly correlated with rs16879248 (r^2^ = 1); alleles:T/C.

e perfectly correlated with rs11868708 (r^2^ = 1); alleles:T/C.

f perfectly correlated with rs11703570 (r^2^ = 1); alleles:C/T.

Finally, given the moderate effects observed in single SNP analysis, we evaluated the joint effect of the 3 genetic risk variants (rs16879248, rs11868708 and rs1173570) that were significant in the single SNP analysis. As shown in *Table 4*, the 3 variants appeared to show a strong cumulative association with ischemic stroke risk. The increased number of the risk alleles was significantly associated with an increased risk of ischemic stroke; the joint effect was independent of aforementioned non-genetic risk factors (*P*_trend_ = 2.0×10^−7^) and remained significant after correction for model selection bias (corrected *P*_trend_ = 0.013, 5000 permutations).

**Table 3:** Haplotype Frequencies Distributions of Significant Haplotype Blocks among Cases and Controls.

Haplotype	Candidate Gene Study							Replication Study						
	SNPs	Allele^g^	Freq		Chi Sq	*P*	*P* h	SNPs	Allele^g^	Freq		Chi Sq	*P*	*P* h
			Case	Control						Case	Control			
*SHMT1*-CCG	rs2273028	C/T	0.364	0.299	6.69	9.71x10^-3^	0.78	rs2273028	C/T	0.339	0.312	1.434	0.23	0.96
	rs11868708	T/C						rs9909104^i^	T/C					
	rs2273026	G/A						rs2273026	G/A					
*TCN2*-GA	rs5749131	A/G	0.042	0.085	11.12	8.54x10^-4^	0.13	rs5749131	A/G	0.063	0.06	0.094	0.76	1
	rs11703570	A/T						rs2301955^j^	C/T					

g Major alleles given first, minor alleles second.

h 1000 permutations performed.

i r^2^ = 1 with rs11868708.

j r^2^ = 1 with rs11703570.

### Replication Study

In the replication study, the allelic ORs of all the 3 tagging SNPs and the haplotype in *SHMT1* and *TCN2* were observed to be in the same direction of effect as the initial study *(* *Table 2* *&* *3* *)*, although these results did not reach statistical significance.

However, the joint association analysis of the 3 variants in the replication study showed a similar cumulative association with ischemic stroke. Although not statistically significant, the trend of elevated stroke risk with an increased number of risk alleles was largely consistent in both studies, with exception of the group with 4 total risk alleles (*Table 4*). This may be attributed to the small size of the subgroups with different numbers of risk alleles. When the initial and replication samples were combined, a clear trend of elevated ORs with increased number of risk alleles was observed and the joint effect was independent of the aforementioned non-genetic risk factors (Combined *P*_trend_ = 1.2×10^−6^).

## Discussion

Stroke is a multifactorial and polygenic disorder with some well-identified environment factors, but the genetic risk factors for stroke remain to be elucidated. In the initial study, we performed a comprehensive analysis of 25 candidate genes involved in the homocysteine metabolic pathway in a well-characterized group of ethnic Chinese with ischemic stroke. We analyzed controls matched for ethnicity and geographic location, thus reducing the possible effects of population stratification on the association results. Moreover, cases and controls were found to be well-matched and of Chinese ancestry in a principle component analysis with 147 tagging SNPs (*Figure S1*). The differences between cases and controls in terms of age, smoking, gender, hypertension and diabetes reflect the established associations of these factors with ischemic stroke; the prevalence of smoking, hypertension and diabetes in controls was similar to the Singapore general population according to the National Health Survey 2004 (NHS 2004). To our knowledge, this is the most comprehensive genetic association study to date of the homocysteine metabolic pathway genes in subjects of Chinese ethnicity with ischemic stroke.

Our initial study provided suggestive association evidence for 3 candidate genes whose genetic effect are likely independent of other common non-genetic risk factors such as age, gender, smoking, hypertension, diabetes and hyperlipidemia. In addition, although the association evidence for the 3 genetic risk variants are moderate and did not survive correction for multiple testing, the joint effect of these variants is strong and the association remained significant after correction for model selection bias.

We did not find a significant association between *MTHFR* C677T and ischemic stroke risk in our initial samples. *MTHFR* C677T has been shown to be associated with ischemic stroke risk in Chinese population by a multi-center case-control study, but the genetic effect was found to be relatively weak (OR = 1.27, TT versus CC homozygotes) [19]. We estimate that our study had only 21% power to detect this weak association.

None of the single SNPs or haplotypes showed statistically significant association in the replication study. With the current sample size of the replication study, we had limited statistical power to validate the small to moderate effects of single SNPs. Moreover, the case and control subjects were 5–8 years younger compared to the initial study, and the inclusion of young population controls carrying risk factors that may contribute to the development of stroke at older ages could have further reduced the power of the replication study. We estimate that our combined study had 90% power (significance level = 0.0005, corrected for 147 tagging SNPs) to detect single SNPs with MAF 0.2 with ORs of 1.5 (*Table S5*). The lack of significant association excludes the presence of common SNPs in the homocysteine metabolic pathways with effect sizes of this magnitude or greater on stroke risk.

Despite the limitations, a trend of elevated ORs with increasing number of total risk alleles was seen in the combined initial and replication samples. This result appears to suggest the 3 variants might each have small to moderate genetic effects that jointly contributed to the association of total number of risk alleles with ischemic stroke risk. This further highlights the importance of joint association analysis of the cumulative effect from multiple variants in detecting small to moderate genetic effects. While the effect size of each positive gene association was weak, the overall contribution of genetic factors from homocysteine metabolic pathway to ischemic stroke risk is likely to be relatively large, with a 5.43-fold different in stroke risk between individuals carrying 5 risk alleles and those carrying 0 or 1 risk alleles. This could also explain the finding that apart from *MTHFR* C677T which has a relatively stronger genetic effect, most of the associations among other investigated candidate genes involved in homocysteine metabolism could not be consistently replicated[20].

**Figure 1:**
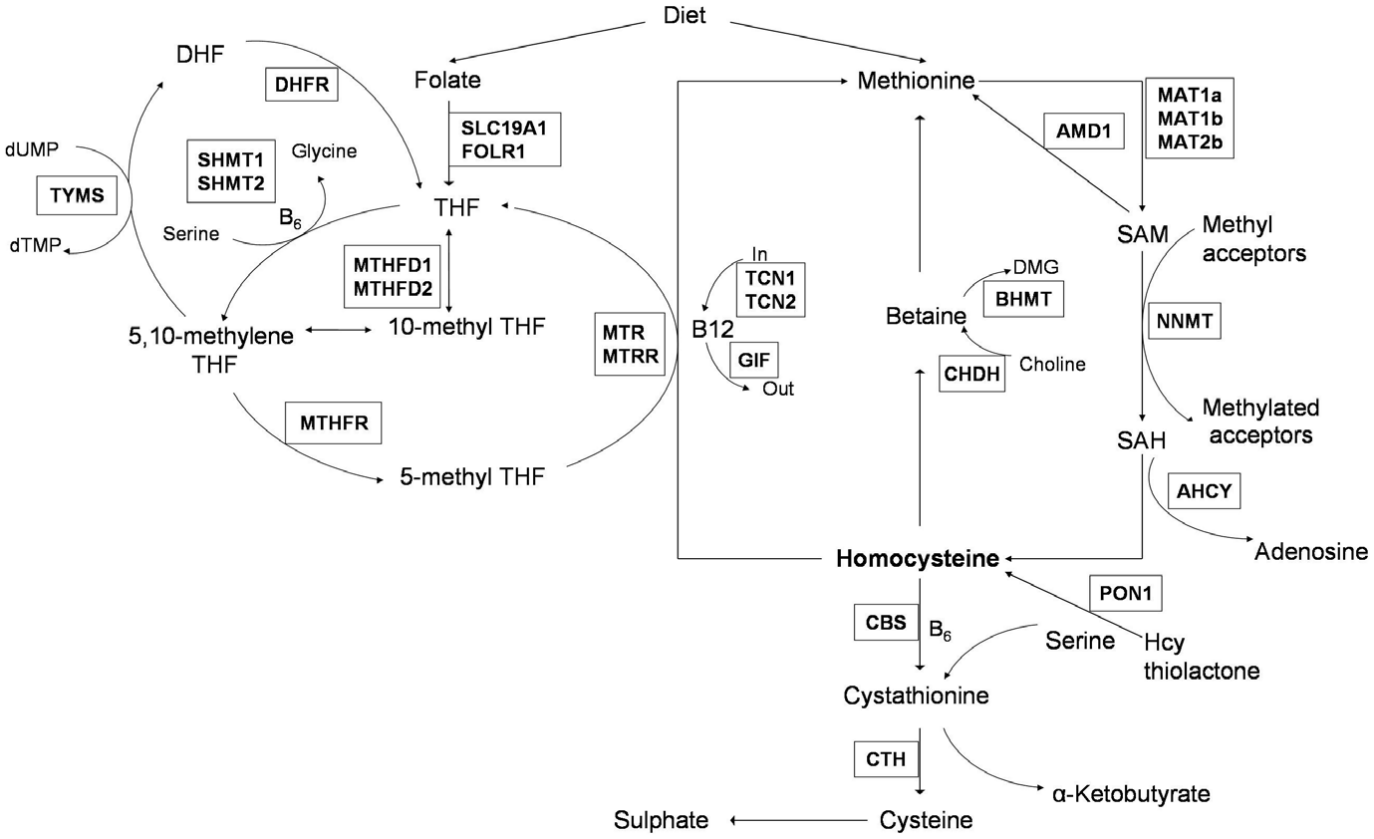
Homocysteine Metabolic Pathways. For gene names, refer to *Table S2*.(Figure adapted from Souto JC, Blanco-Vaca F, Soria JM, Buil A, Almasy L, et al. (2005) A genomewide exploration suggests a new candidate gene at chromosome 11q23 as the major determinant of plasma homocysteine levels: results from the GAIT project. Am J Hum Genet 76: 925-933.)

We postulate that these genetic risk variants might affect ischemic stroke risk by jointly modulating homocysteine levels, but further study will be needed to prove this hypothesis. Interestingly, genetic association evidence observed in this study is concentrated in genes that are involved in regulating either folate cycling (*SHMT1* and *MTRR*) or transportation of vitamin B12 (*TCN2*) (*Figure 1*), suggesting the importance of cellular availability of folate and vitamin B12 in influencing ischemic stroke risk. Plasma homocysteine levels are inversely related to plasma concentrations of folate and vitamin B12 as well as to the intake of these vitamins[21]. Recently, vitamin supplements have received much attention as a therapeutic strategy for vascular diseases although the beneficial effects of lowering plasma homocysteine level by vitamins on the risk of ischemic stroke have not yet been established[22]. Our study suggested that deficiency of both folate and vitamin B12 levels, caused by a combination of genetic variations at *SHMT1*, *MTRR* and *TCN2,* may jointly increase ischemic stroke risk. Unfortunately, lack of data on the levels of homocysteine, folate and vitamin B12 in our study subjects limited further investigation of the effect of these genetic variants on ischemic stroke, which could otherwise be further established as supporting evidence for the effect of these variants on the biomarkers.

In conclusion, we have completed a comprehensive genetic association study of the homocysteine metabolic pathway in subjects of Chinese ethnicity. Although none of the studied single variants or haplotypes from the homocysteine metabolic pathway is significantly associated with ischemic stroke risk, our results suggest that the joint effect of several small to moderate risk variants could confer an increase in ischemic stroke risk. We propose that the genetic etiology of ischemic stroke is likely to be complex with many loci modulating homocysteine metabolism and each conferring a small to moderate risk. Further work, such as validation in larger sample sets, is necessary to confirm our findings and to explore the joint effects of additional risk variants.

## Materials and Methods

### Ethics Statement

All participants agreed to provide blood samples and written informed consent for the study. The study was approved by the Singapore General Hospital Ethics Committee and performed in observance of the local institutional guidelines.

### Study Subjects

The initial study recruited 384 stroke patients and 362 non-stroke controls 40 to 85 years of age of Singaporean Chinese ethnicity. All the cases of ischemic stroke were patients admitted to the Singapore General Hospital (SGH) Stroke Programme. Clinical diagnosis and classification of ischemic stroke was confirmed in all patients through the use of brain computerized tomography (CT) or magnetic resonance imaging (MRI). CT or MRI was performed within 48 hours of admission and the patients were systemically evaluated to rigorously determine stroke phenotype and vascular risk factors (hypertension, diabetes, hyperlipidemia and smoking). Demographic (age, gender, and ethnicity) information was also obtained from all the patients. Controls were healthy volunteers from the Singapore population who agreed to provide their demographic, health (weight, height, physical activity) and vascular risk factor information for the study. Stroke was excluded on the basis of family history and a medical examination.

For both cases and controls: hypertension was defined as having a resting blood pressure ≥ 140/90 mmHg, or treatment with anti-hypertensive medications; subjects with diabetes were those having fasting hyperglycemia of ≥ 7.0 mmol**/**L, or treatment with insulin or oral hypoglycemic medications; hyperlipidemia was defined as having a fasting total cholesterol level of ≥ 6.2 mmol/L or treatment with cholesterol lowering medications; smoking was defined as those who previously smoked or those who currently smoked at least 1 cigarette per day.

In the validation study, an additional 708 new stroke samples from the SGH Stroke Programme were recruited. Clinical diagnosis, ischemic stroke classification and demographic information were obtained as described above in the initial study. 903 stroke-free controls were recruited as part of the Singapore Prospective Study Program (SP2) which consists of individuals from 2 previous cross sectional studies, the 1992 National Health Survey and the 1998 National Health survey, each representing a random sample of the Singapore population, were re-contacted between 2004 and 2007 [23]–[24]. Subjects who were successfully re-contacted and gave informed consent answered a questionnaire and attended a clinic examination. Fasting blood glucose was measured for all participants. We observed a significant difference in mean age of 708 cases (61.6±11.7 years) and 903 controls (46.5±10.2 years). Hence, cases and controls were matched with age and 420 case-control pairs were included in the analysis. Samples analyzed in the validation study are independent from the initial samples.

*Table 1* summarizes the characteristics of stroke patients and control subjects in each study. The cases and controls of both the initial and validation cohorts were significantly different in the prevalence of smokers, hypertension and diabetes; to adjust for these differences, we entered these non-genetic factors as covariates in a logistic regression test for association.

**Table 4:** Cumulative Effect of Risk Alleles on Ischemic Stroke Risk.

	Initial Study Study				Replication Study				Combined Analysis			
No of Risk Allele^k^	Ctrl:Case (Number)	OR^l^	*P* m	*P*_Trend_ n	Ctrl:Case (Number)	OR^l^	*P* m	*P*_Trend_ n	Ctrl:Case (Number)	OR^l^	*P* m	*P*_Trend_ n
0 or 1	63:32	1.00	-	2.0x10^-7^	54:38	1.00	-	0.111	117:70	1.00	-	1.20x10^-6^
2	122:111	1.84(1.07-3.15)	0.027		150:142	1.81(0.98-3.32)	0.056		272:253	1.77(1.21-2.60)	0.004	
3	120:134	2.40(1.41-4.08)	0.001		139:155	2.15(1.18-3.94)	0.013		259:289	2.19(1.50-3.21)	5.6x10^-5^	
4	44:66	3.58(1.93-6.67)	5.6x10^-5^		64:65	1.49(0.75-2.95)	0.257		108:131	2.23(1.44-3.46)	3.2x10^-4^	
5	6:17	13.5(3.94-46.30)	3.5x10^-5^		13:20	3.16(1.16-8.60)	0.025		19:37	5.43(2.68-11.00)	2.7x10^-6^	

k Risk alleles are rs16879248:T or rs16879259:T, rs11868708:C or rs9909104:C, rs11703570:A or rs2301955:T.

l Adjusted risk factors: gender, age, hypertension, diabetes, hyperlipidemia, smoking.

m Significance by comparing to the base group carrying 0 or 1 risk allele.

n *P* value calculated by Cochran-Armitage Trends Test from the adjusted ORs.

### SNP Selection and Genotyping

25 candidate genes known to be involved in homocysteine metabolic pathway (*Figure 1*, *Table S2*) were selected for the present study. The selection of the SNPs was done by using the SNP information from the International HapMap project (Phase I data) [25] and the dbSNP database (Build 124) to produce an evenly distributed set of SNPs for each gene. In the initial study, 417 SNPs from the 25 candidate genes were selected for genotyping analysis using the MassArray system from Sequenom (San Diego, USA) and the GoldenGate Assay from Illumina (San Diego, USA). After quality control filtering (criteria: MAF>0.03, call rate>0.9, HWE P>0.05), the final dataset for association analysis contains the genotyping results of 285 SNPs in 714 samples. In the replication study, 8 SNPs which show significant association in the SNP-based and haplotype analysis were selected and genotyped using MassArray system from Sequenom (San Diego, USA) (*Table S3*). More information on the procedure of SNP selection is provided in the *Text S1*.

### Statistical Analyses

All statistical analyses were performed using STATA 8.0 (StataCorp, TX: College Station), Haploview (version 4.0) and R program (version 2.2.1) [26]–[27]. To reduce the redundancy of SNPs in association analysis and thus the number of association tests, 147 tagging SNPs were identified from the 285 SNPs by using Paul de Bakker' Tagger tag SNP selection algorithm implemented in Haploview and an r^2^ cut-off of 0.8[26]. Association analysis was then performed on the tagging SNPs using the Cochrane-Armitage trend test. Genetic association was expressed as odds ratios (OR) of the rarer allele versus the common allele for having the disease phenotype, and the significance of association was tested using a logistic regression analysis adjusting for vascular risk factors, age and gender.

Haplotype analysis was performed within the regions showing strong linkage disequilibrium (LD). Haplotype phase and population frequency were inferred and estimated from genotypes using the Expectation-Maximization (EM) algorithm implemented in Haploview, and 35 LD blocks within 19 genes were identified using the Confidence Intervals method[28]-[29]. Haplotype association analysis was then performed for each common haplotype (population frequency above 0.01) using Haploview. For haplotypes showing suggestive evidence (*P*<0.05) by Haploview analysis, PHASE (v2.1) was used to reconstruct the haplotypes from the genotypes[30]–[31]. In order to account for the multiple SNPs and haplotypes tested, a permutation procedure (implemented in the Haploview) was used to obtain the corrected *P* value of haplotype association.

To assess cumulative effect of multiple variants, a joint association analysis of the SNPs showing suggestive evidence (*P*<0.05) in single SNP association testing was performed. The total number of risk alleles of the 3 SNPs in each individual was counted in each individual, as done in a recent study on prostate cancer[32]. Given the small number of individuals carrying none of the risk alleles, ORs of individuals carrying 2,3,4 and 5 risk alleles over the ones carrying none or 1 risk allele for developing ischemic stroke were evaluated using logistic regression analysis with adjustment for the aforementioned risk factors. To correct for model selection bias of the joint analysis, 5000 permutations were performed to assess significance of the joint effect. Permutation was done by shuffling the phenotype status in the 714 samples. In each permutation, SNPs with suggestive evidence of association (adjusted *P*<0.05) from the trend analysis were identified first, and significance (*P* value) of the joint effect of suggested SNPs was then evaluated in the same way using multi-variable logistic regression analysis. Finally, *P* values from 5000 permutations were used to determine significance or exact *P* value of the joint effect of the 3 risk variants identified in this study.

Pair-wise interaction among SNPs was also investigated by adding an interaction term into the logistic regression analysis. An interaction is found to be significant if likelihood-ratio test *P* value is less than 0.05. All power estimations were computed using QUANTO (v1.2) [33]–[34].

SNP-based, haplotype and joint association analysis for the 8 selected SNPs in replication samples were carried out as described above.

## Supporting Information

Figure S1 **Principal Component Analysis (PCA) of the Initial Samples.** Plots between the 1st and the 2nd-5th PCs, which were derived from the PCA analysis of 714 initial samples and 194 HapMap samples. All the cases are labeled in red, whereas the controls are labeled in white. The HapMap samples are labeled in blue (CHB), earth yellow (JPT), yellow (CEU) and green (YRI).
(DOCX)

Table S1 **Cochran-Armitage Trends Test Result for the 147 Tagging SNPs.**
(DOCX)

Table S2 **Number of SNPs Analyzed for each Candidate Gene.**
(DOCX)

Table S3 **SNPs selected for replication study.**
(DOCX)

Table S4 **Association of 3 Significant SNPs with Ischemic Stroke Susceptibility under Different Inheritance Models.**
(DOCX)

Table S5 **Power Calculation for Two-stage Case-Control Association Study (Stage 1: 360 Cases & 354 Controls; Stage 2: 420 Cases & 420 Controls) at a Prevalence of 3% (Significant Level = 0.0005, 147 SNPs).**
(DOCX)

Text S1 **Supplementary information of method.**
(DOCX)
