# Supplementary figures and images for: A Comprehensive Association Analysis of Homocysteine Metabolic Pathway Genes in Singaporean Chinese with Ischemic Stroke
Source: PLoS One. 2011 Sep 15;6(9):e24757. doi: 10.1371/journal.pone.0024757 (PMC3174208; doi:10.1371/journal.pone.0024757)

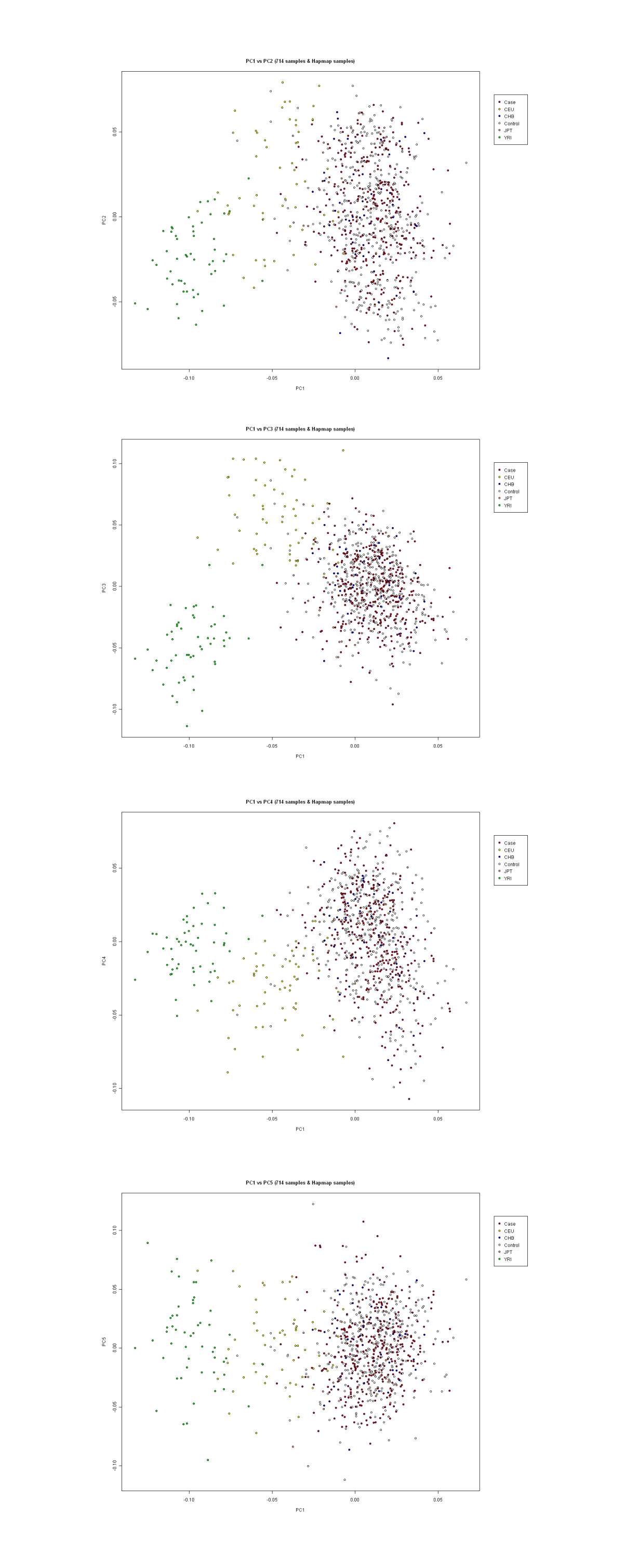


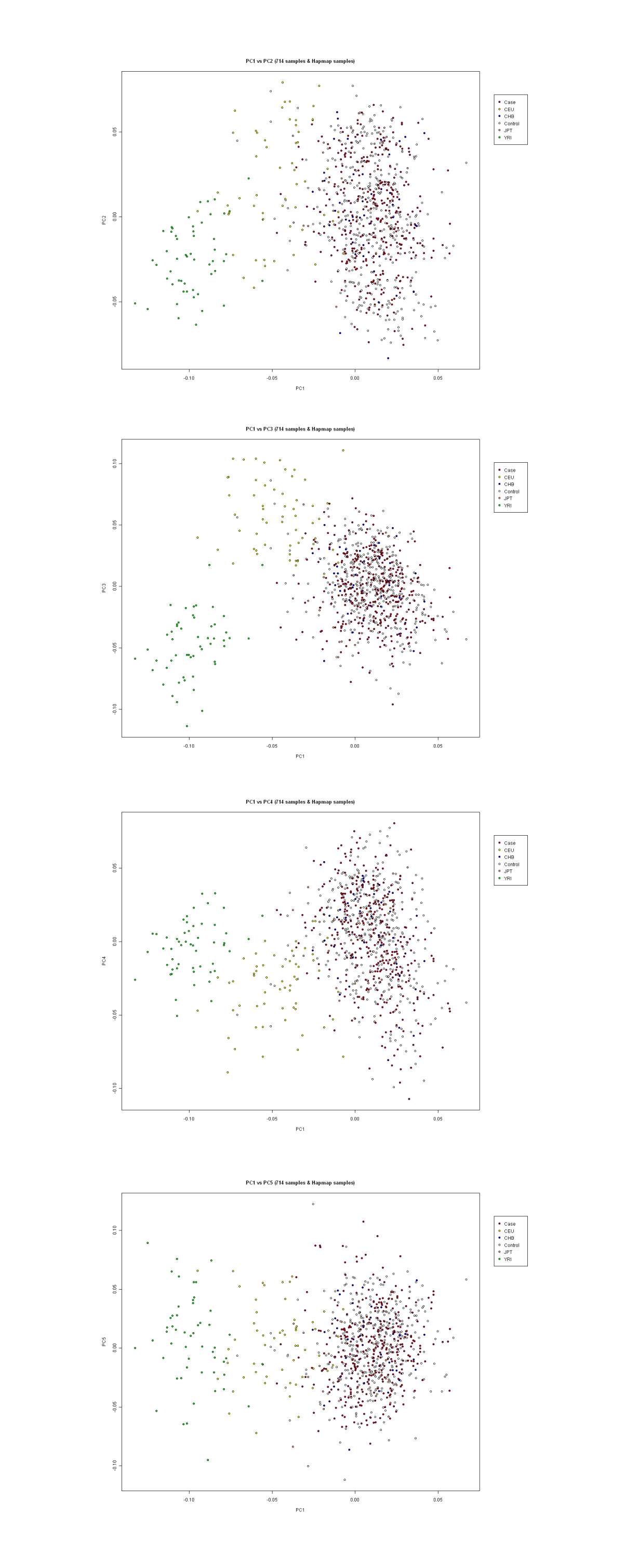

Supplement: Figure S1 — Principal Component Analysis (PCA) of the Initial Samples. Plots between the 1st and the 2nd-5th PCs, which were derived from the PCA analysis of 714 initial samples and 194 HapMap samples. All the cases are labeled in red, whereas the controls are labeled in white. The HapMap samples are labeled in blue (CHB), earth yellow (JPT), yellow (CEU) and green (YRI). (DOCX) [file pone.0024757.s001.docx]
