# Supplementary material for: A Comprehensive Association Analysis of Homocysteine Metabolic Pathway Genes in Singaporean Chinese with Ischemic Stroke
Source: PLoS One. 2011 Sep 15;6(9):e24757. doi: 10.1371/journal.pone.0024757 (PMC3174208; doi:10.1371/journal.pone.0024757)
Supplement: Table S1 — Cochran-Armitage Trends Test Result for the 147 Tagging SNPs. (DOCX) [file pone.0024757.s002.docx]

**SUPPLEMENTARY TABLE 1, Cochran-Armitage** **Trends Test Result for the 147 Tagging SNPs.**

| SNP | Gene | Trends of Odd (p-value) | OR_AB_/OR_AA_ | 95% CI | | | OR_BB_/OR_AA_ | 95% CI | | |
| --- | --- | --- | --- | --- | --- | --- | --- | --- | --- | --- |
| AHCY | rs819175 | 0.3942 | 1.09 | 0.80 | - | 1.49 | 1.32 | 0.64 | - | 2.73 |
| AMD1 | rs1007274 | 0.2234 | 1.16 | 0.85 | - | 1.59 | 1.38 | 0.70 | - | 2.72 |
| AMD1 | rs1279597 | 0.3843 | 1.04 | 0.77 | - | 1.42 | 1.35 | 0.77 | - | 2.38 |
| AMD1 | rs2796749 | 0.629 | 1.01 | 0.74 | - | 1.39 | 0.85 | 0.53 | - | 1.37 |
| AMD1 | rs9400425 | 0.6164 | 0.79 | 0.58 | - | 1.08 | 1.08 | 0.65 | - | 1.79 |
| BHMT | rs3797546 | 0.4822 | 0.97 | 0.71 | - | 1.32 | 1.38 | 0.80 | - | 2.39 |
| BHMT | rs4421087 | 0.2729 | 0.83 | 0.61 | - | 1.14 | 0.84 | 0.35 | - | 2.02 |
| BHMT | rs506500 | 0.7155 | 1.01 | 0.65 | - | 1.56 | - |  | - |  |
| BHMT | rs567754 | 0.4238 | 1.01 | 0.72 | - | 1.43 | 1.19 | 0.79 | - | 1.80 |
| BHMT | rs600473 | 0.7563 | 1.04 | 0.77 | - | 1.42 | 0.80 | 0.44 | - | 1.46 |
| BHMT | rs651852 | 0.3281 | 1.01 | 0.74 | - | 1.39 | 0.72 | 0.44 | - | 1.17 |
| BHMT | rs7700970 | 0.4151 | 0.86 | 0.57 | - | 1.29 | 0.77 | 0.20 | - | 2.88 |
| BHMT | rs955897 | 0.9889 | 1.06 | 0.76 | - | 1.47 | 0.98 | 0.64 | - | 1.49 |
| CBS | rs1051319 | 0.4883 | 1.16 | 0.79 | - | 1.70 | 1.01 | 0.20 | - | 5.06 |
| CBS | rs2014564 | 0.6934 | 1.24 | 0.91 | - | 1.70 | 0.88 | 0.52 | - | 1.49 |
| CBS | rs234709 | 0.3993 | 1.19 | 0.83 | - | 1.69 | 1.02 | 0.29 | - | 3.58 |
| CBS | rs2849727 | 0.5936 | 1.18 | 0.84 | - | 1.63 | 1.08 | 0.70 | - | 1.66 |
| CBS | rs2849731 | 0.7167 | 1.17 | 0.85 | - | 1.60 | 0.76 | 0.47 | - | 1.25 |
| CBS | rs706209 | 0.7976 | 1.30 | 0.94 | - | 1.81 | 0.97 | 0.63 | - | 1.49 |
| CBS | rs9977307 | 0.7771 | 1.11 | 0.81 | - | 1.52 | 0.84 | 0.53 | - | 1.35 |
| CHDH | rs11130381 | 0.555 | 1.06 | 0.74 | - | 1.50 | 0.87 | 0.57 | - | 1.34 |
| CHDH | rs12496647 | 0.7142 | 0.95 | 0.69 | - | 1.30 | 1.47 | 0.69 | - | 3.14 |
| CHDH | rs13317328 | 0.792 | 1.23 | 0.89 | - | 1.69 | 0.83 | 0.53 | - | 1.31 |
| CHDH | rs2289209 | 0.2697 | 1.13 | 0.77 | - | 1.65 | 1.83 | 0.61 | - | 5.55 |
| CHDH | rs4687747 | 0.3685 | 1.11 | 0.79 | - | 1.57 | 1.46 | 0.55 | - | 3.89 |
| CHDH | rs7620929 | 0.7312 | 1.21 | 0.85 | - | 1.73 | 1.06 | 0.69 | - | 1.63 |
| CHDH | rs7626693 | 0.9568 | 0.83 | 0.60 | - | 1.16 | 1.09 | 0.71 | - | 1.69 |
| CHDH | rs7627178 | 0.2467 | 1.50 | 1.05 | - | 2.14 | 1.24 | 0.81 | - | 1.91 |
| CHDH | rs877484 | 0.5815 | 0.86 | 0.63 | - | 1.18 | 0.99 | 0.57 | - | 1.71 |
| CHDH | rs930367 | 0.5462 | 0.95 | 0.70 | - | 1.30 | 0.85 | 0.50 | - | 1.43 |
| CTH | rs1021737 | 0.7908 | 1.03 | 0.75 | - | 1.42 | 1.09 | 0.44 | - | 2.74 |
| CTH | rs1145920 | 0.1528 | 1.31 | 0.96 | - | 1.80 | 1.08 | 0.44 | - | 2.64 |
| CTH | rs12133065 | 0.5847 | 0.87 | 0.64 | - | 1.18 | 1.07 | 0.51 | - | 2.25 |
| CTH | rs4650050 | 0.7175 | 1.13 | 0.83 | - | 1.54 | 0.98 | 0.57 | - | 1.65 |
| CTH | rs535112 | 0.347 | 0.88 | 0.65 | - | 1.19 | 0.79 | 0.36 | - | 1.76 |
| CTH | rs681475 | 0.0817 | 0.82 | 0.57 | - | 1.19 | 0.67 | 0.43 | - | 1.05 |
| DHFR | rs1677667 | 0.2203 | 0.76 | 0.47 | - | 1.25 | 0.48 | 0.04 | - | 5.30 |
| DHFR | rs2431221 | 0.0255 | 0.56 | 0.33 | - | 0.95 | 0.47 | 0.04 | - | 5.17 |
| FOLR1 | rs11235462 | 0.3521 | 0.71 | 0.51 | - | 0.98 | 0.95 | 0.60 | - | 1.52 |
| FOLR1 | rs2071010 | 0.5056 | 0.93 | 0.67 | - | 1.29 | 0.68 | 0.21 | - | 2.19 |
| FOLR1 | rs3016432 | 0.2742 | 0.63 | 0.44 | - | 0.88 | 0.85 | 0.56 | - | 1.31 |
| FOLR1 | rs7109250 | 0.4077 | 1.31 | 0.87 | - | 1.97 | 0.50 | 0.09 | - | 2.77 |
| GIF | rs17596862 | 0.5928 | 1.17 | 0.66 | - | 2.08 | - |  | - |  |
| GIF | rs4356243 | 0.4745 | 1.21 | 0.72 | - | 2.02 | - |  | - |  |
| GIF | rs558660 | 0.8247 | 0.82 | 0.60 | - | 1.13 | 1.09 | 0.68 | - | 1.72 |
| MAT1A | rs1010245 | 0.6748 | 1.21 | 0.71 | - | 2.06 | - |  | - |  |
| MAT1A | rs10887708 | 0.3137 | 0.76 | 0.54 | - | 1.06 | 0.84 | 0.56 | - | 1.28 |
| MAT1A | rs2236569 | 0.481 | 0.93 | 0.60 | - | 1.43 | - |  | - |  |
| MAT1A | rs9285726 | 0.2628 | 1.41 | 0.77 | - | 2.59 | - |  | - |  |
| MAT2A | rs2028898 | 0.5504 | 0.96 | 0.70 | - | 1.31 | 0.86 | 0.53 | - | 1.40 |
| MAT2A | rs3755015 | 0.3736 | 1.03 | 0.75 | - | 1.42 | 0.77 | 0.50 | - | 1.20 |
| MAT2B | rs10035535 | 0.692 | 1.08 | 0.78 | - | 1.50 | 1.01 | 0.35 | - | 2.92 |
| MAT2B | rs1363730 | 0.3276 | 1.29 | 0.79 | - | 2.11 | 1.01 | 0.06 | - | 16.24 |
| MAT2B | rs4869087 | 0.3404 | 1.22 | 0.82 | - | 1.80 | 1.02 | 0.06 | - | 16.38 |
| MAT2B | rs6874065 | 0.3922 | 1.15 | 0.84 | - | 1.56 | 1.15 | 0.46 | - | 2.90 |
| MTHFD1 | rs11158538 | 0.8262 | 1.06 | 0.75 | - | 1.50 | 1.00 | 0.45 | - | 2.19 |
| MTHFD1 | rs11622591 | 0.0707 | 1.49 | 1.08 | - | 2.06 | 1.05 | 0.50 | - | 2.17 |
| MTHFD1 | rs1256142 | 0.8055 | 1.14 | 0.80 | - | 1.61 | 0.94 | 0.62 | - | 1.41 |
| MTHFD1 | rs1256146 | 0.48 | 0.87 | 0.61 | - | 1.24 | 0.95 | 0.19 | - | 4.77 |
| MTHFD1 | rs17824591 | 0.6014 | 0.85 | 0.47 | - | 1.55 | - |  | - |  |
| MTHFD1 | rs1885031 | 0.5609 | 1.17 | 0.85 | - | 1.60 | 1.07 | 0.67 | - | 1.69 |
| MTHFD1 | rs1950902 | 0.4906 | 1.13 | 0.82 | - | 1.55 | 1.12 | 0.71 | - | 1.78 |
| MTHFD1 | rs1956545 | 0.7785 | 0.94 | 0.67 | - | 1.33 | 1.07 | 0.71 | - | 1.62 |
| MTHFD1 | rs2236222 | 0.1003 | 1.36 | 1.00 | - | 1.86 | 1.22 | 0.66 | - | 2.24 |
| MTHFD1 | rs2295640 | 0.3873 | 1.33 | 0.96 | - | 1.84 | 1.11 | 0.72 | - | 1.72 |
| MTHFD1 | rs3783726 | 0.946 | 1.24 | 0.91 | - | 1.69 | 0.79 | 0.46 | - | 1.33 |
| MTHFD1 | rs3783728 | 0.8964 | 1.04 | 0.65 | - | 1.66 | 0.96 | 0.13 | - | 6.90 |
| MTHFD1 | rs4902281 | 0.9019 | 1.05 | 0.76 | - | 1.44 | 0.93 | 0.59 | - | 1.47 |
| MTHFD1 | rs8013633 | 0.1709 | 1.29 | 0.95 | - | 1.77 | 1.22 | 0.73 | - | 2.02 |
| MTHFD1 | rs9323450 | 0.2524 | 0.78 | 0.55 | - | 1.09 | 0.81 | 0.53 | - | 1.24 |
| MTHFD2 | rs10199560 | 0.6424 | 0.91 | 0.62 | - | 1.33 | 0.97 | 0.19 | - | 4.83 |
| MTHFD2 | rs13385639 | 0.7654 | 1.07 | 0.71 | - | 1.62 | 0.99 | 0.14 | - | 7.11 |
| MTHFD2 | rs13426653 | 0.2443 | 1.10 | 0.80 | - | 1.52 | 1.49 | 0.77 | - | 2.90 |
| MTHFD2 | rs1667627 | 0.4261 | 0.92 | 0.67 | - | 1.27 | 0.84 | 0.54 | - | 1.31 |
| MTHFD2 | rs828858 | 0.162 | 1.28 | 0.88 | - | 1.86 | 1.46 | 0.46 | - | 4.67 |
| MTHFR | rs10864542 | 0.3456 | 1.22 | 0.89 | - | 1.66 | 1.06 | 0.55 | - | 2.03 |
| MTHFR | rs11586659 | 0.6247 | 1.12 | 0.76 | - | 1.64 | 1.00 | 0.29 | - | 3.51 |
| MTHFR | rs1537514 | 0.5194 | 1.08 | 0.76 | - | 1.53 | 1.49 | 0.41 | - | 5.34 |
| MTHFR | rs1801133 | 0.9182 | 0.89 | 0.65 | - | 1.22 | 1.41 | 0.71 | - | 2.80 |
| MTHFR | rs1994798 | 0.0482 | 1.26 | 0.92 | - | 1.72 | 1.69 | 0.89 | - | 3.21 |
| MTR | rs10158822 | 0.0939 | 1.24 | 0.87 | - | 1.77 | 3.14 | 0.62 | - | 15.74 |
| MTR | rs1050999 | 0.7398 | 1.20 | 0.87 | - | 1.66 | 1.00 | 0.65 | - | 1.54 |
| MTR | rs10733118 | 0.2703 | 1.21 | 0.87 | - | 1.68 | 0.71 | 0.47 | - | 1.09 |
| MTR | rs10802564 | 0.2009 | 0.99 | 0.70 | - | 1.40 | 0.76 | 0.50 | - | 1.14 |
| MTR | rs12138911 | 0.2439 | 1.12 | 0.79 | - | 1.59 | 1.64 | 0.70 | - | 3.88 |
| MTR | rs12562226 | 0.764 | 0.98 | 0.62 | - | 1.55 | 0.49 | 0.04 | - | 5.43 |
| MTR | rs2275565 | 0.2535 | 1.11 | 0.80 | - | 1.55 | 1.78 | 0.69 | - | 4.62 |
| MTR | rs2275568 | 0.8425 | 1.20 | 0.86 | - | 1.67 | 1.00 | 0.66 | - | 1.51 |
| MTR | rs4659724 | 0.76 | 1.07 | 0.78 | - | 1.47 | 1.04 | 0.66 | - | 1.64 |
| MTR | rs4659726 | 0.3685 | 1.13 | 0.82 | - | 1.56 | 0.43 | 0.21 | - | 0.91 |
| MTR | rs7367859 | 0.3024 | 1.12 | 0.81 | - | 1.55 | 0.74 | 0.48 | - | 1.13 |
| MTR | rs7541539 | 0.5072 | 0.88 | 0.65 | - | 1.20 | 0.93 | 0.49 | - | 1.79 |
| MTRR | rs1532268 | 0.3925 | 1.16 | 0.83 | - | 1.61 | 1.14 | 0.46 | - | 2.86 |
| MTRR | rs161871 | 0.2742 | 0.96 | 0.70 | - | 1.32 | 0.75 | 0.47 | - | 1.18 |
| MTRR | rs162031 | 0.2064 | 1.18 | 0.85 | - | 1.66 | 1.30 | 0.85 | - | 1.99 |
| MTRR | rs162035 | 0.4824 | 0.81 | 0.58 | - | 1.15 | 1.13 | 0.50 | - | 2.58 |
| MTRR | rs16879248 | 0.0039 | 0.72 | 0.53 | - | 0.98 | 0.37 | 0.16 | - | 0.88 |
| MTRR | rs16879258 | 0.8861 | 1.16 | 0.70 | - | 1.93 | - |  | - |  |
| MTRR | rs326121 | 0.3087 | 1.10 | 0.80 | - | 1.50 | 1.28 | 0.79 | - | 2.08 |
| MTRR | rs3822444 | 0.3051 | 1.04 | 0.76 | - | 1.43 | 0.41 | 0.17 | - | 0.95 |
| MTRR | rs8659 | 0.3267 | 0.89 | 0.65 | - | 1.22 | 0.80 | 0.49 | - | 1.32 |
| NNMT | rs11214921 | 0.8254 | 0.98 | 0.71 | - | 1.36 | 0.95 | 0.62 | - | 1.47 |
| NNMT | rs11214934 | 0.6894 | 1.09 | 0.78 | - | 1.50 | 0.87 | 0.56 | - | 1.34 |
| NNMT | rs11569688 | 0.6586 | 0.83 | 0.49 | - | 1.39 | - |  | - |  |
| NNMT | rs17116804 | 0.6153 | 1.07 | 0.60 | - | 1.91 | - |  | - |  |
| NNMT | rs1941399 | 0.2011 | 1.20 | 0.87 | - | 1.67 | 1.40 | 0.58 | - | 3.39 |
| NNMT | rs1941404 | 0.3077 | 0.90 | 0.63 | - | 1.28 | 0.80 | 0.52 | - | 1.23 |
| NNMT | rs2511153 | 0.5776 | 1.18 | 0.86 | - | 1.63 | 1.06 | 0.67 | - | 1.67 |
| NNMT | rs694539 | 0.8282 | 0.95 | 0.70 | - | 1.30 | 1.12 | 0.69 | - | 1.82 |
| PON1 | rs2074354 | 0.5604 | 1.15 | 0.82 | - | 1.61 | 1.11 | 0.72 | - | 1.72 |
| PON1 | rs2237584 | 0.4998 | 1.08 | 0.79 | - | 1.47 | 0.69 | 0.40 | - | 1.19 |
| PON1 | rs2299257 | 0.4708 | 0.96 | 0.70 | - | 1.32 | 0.83 | 0.52 | - | 1.32 |
| PON1 | rs2299260 | 0.4309 | 0.99 | 0.69 | - | 1.43 | 0.36 | 0.09 | - | 1.38 |
| PON1 | rs3917468 | 0.9335 | 0.97 | 0.70 | - | 1.34 | 1.04 | 0.51 | - | 2.10 |
| PON1 | rs3917548 | 0.4312 | 0.96 | 0.67 | - | 1.39 | 0.32 | 0.06 | - | 1.61 |
| PON1 | rs705381 | 0.4613 | 1.12 | 0.78 | - | 1.62 | 1.51 | 0.25 | - | 9.13 |
| PON1 | rs757158 | 0.7168 | 0.96 | 0.68 | - | 1.37 | 0.92 | 0.60 | - | 1.42 |
| PON1 | rs854544 | 0.8958 | 1.02 | 0.75 | - | 1.40 | 0.94 | 0.59 | - | 1.52 |
| PON2 | rs854548 | 0.9721 | 0.95 | 0.68 | - | 1.31 | 1.14 | 0.59 | - | 2.19 |
| PON3 | rs854551 | 0.5865 | 1.04 | 0.70 | - | 1.54 | - |  | - |  |
| SHMT1 | rs11868708 | 0.0096 | 1.29 | 0.94 | - | 1.76 | 1.88 | 1.13 | - | 3.14 |
| SHMT1 | rs2273026 | 0.0591 | 0.75 | 0.53 | - | 1.05 | 0.59 | 0.21 | - | 1.69 |
| SHMT1 | rs2273027 | 0.5483 | 1.20 | 0.84 | - | 1.71 | 1.13 | 0.74 | - | 1.72 |
| SHMT1 | rs2273028 | 0.6232 | 0.97 | 0.64 | - | 1.49 | 0.32 | 0.03 | - | 3.15 |
| SHMT1 | rs669340 | 0.5511 | 0.96 | 0.69 | - | 1.34 | 0.87 | 0.57 | - | 1.34 |
| SHMT2 | rs10783816 | 0.9979 | 1.18 | 0.83 | - | 1.69 | 1.00 | 0.66 | - | 1.51 |
| SLC19A1 | rs1023159 | 0.9779 | 0.89 | 0.65 | - | 1.21 | 1.22 | 0.69 | - | 2.14 |
| SLC19A1 | rs12659 | 0.763 | 0.90 | 0.64 | - | 1.28 | 0.95 | 0.62 | - | 1.46 |
| SLC19A1 | rs13050920 | 0.9382 | 1.07 | 0.76 | - | 1.51 | 1.01 | 0.66 | - | 1.54 |
| SLC19A1 | rs17004785 | 0.968 | 0.99 | 0.61 | - | 1.62 | 0.97 | 0.13 | - | 6.91 |
| SLC19A1 | rs2330184 | 0.8488 | 0.98 | 0.72 | - | 1.34 | 0.95 | 0.55 | - | 1.65 |
| SLC19A1 | rs2838957 | 0.9166 | 0.96 | 0.67 | - | 1.37 | 0.98 | 0.64 | - | 1.50 |
| SLC19A1 | rs3788190 | 0.8291 | 0.96 | 0.69 | - | 1.34 | 0.96 | 0.62 | - | 1.48 |
| TCN1 | rs17154234 | 0.9612 | 0.98 | 0.48 | - | 2.00 | - |  | - |  |
| TCN1 | rs526934 | 0.5809 | 0.84 | 0.61 | - | 1.16 | 1.15 | 0.54 | - | 2.45 |
| TCN2 | rs11703570 | 0.0433 | 1.38 | 1.00 | - | 1.89 | 1.46 | 0.60 | - | 3.55 |
| TCN2 | rs2267163 | 0.3414 | 1.29 | 0.93 | - | 1.78 | 1.15 | 0.75 | - | 1.76 |
| TCN2 | rs2283873 | 0.8715 | 1.02 | 0.74 | - | 1.40 | 1.05 | 0.52 | - | 2.14 |
| TCN2 | rs5749131 | 0.4986 | 0.93 | 0.68 | - | 1.27 | 0.83 | 0.45 | - | 1.54 |
| TCN2 | rs7289549 | 0.1706 | 1.10 | 0.80 | - | 1.52 | 1.38 | 0.88 | - | 2.17 |
| TYMS | rs12373391 | 0.7543 | 1.09 | 0.78 | - | 1.52 | 1.05 | 0.68 | - | 1.61 |
| TYMS | rs2298582 | 0.8287 | 1.03 | 0.69 | - | 1.54 | - |  | - |  |
| TYMS | rs2853741 | 0.8495 | 1.11 | 0.78 | - | 1.56 | 0.95 | 0.63 | - | 1.43 |
| TYMS | rs3826626 | 0.5201 | 1.08 | 0.80 | - | 1.48 | 1.19 | 0.59 | - | 2.40 |
| TYMS | rs502396 | 0.2748 | 1.31 | 0.96 | - | 1.79 | 1.05 | 0.61 | - | 1.82 |
| TYMS | rs523230 | 0.5321 | 1.04 | 0.73 | - | 1.47 | 0.86 | 0.56 | - | 1.32 |
| TYMS | rs699517 | 0.3601 | 1.15 | 0.84 | - | 1.57 | 0.66 | 0.41 | - | 1.07 |
