# Supplementary material for: A Comprehensive Association Analysis of Homocysteine Metabolic Pathway Genes in Singaporean Chinese with Ischemic Stroke
Source: PLoS One. 2011 Sep 15;6(9):e24757. doi: 10.1371/journal.pone.0024757 (PMC3174208; doi:10.1371/journal.pone.0024757)
Supplement: Table S2 — Number of SNPs Analyzed for each Candidate Gene. (DOCX) [file pone.0024757.s003.docx]

| Gene | Chr | Size(Kb) | Full Name | Number of SNPs | | | | | Tagging SNPs for Association Analysis |
| --- | --- | --- | --- | --- | --- | --- | --- | --- | --- |
|  |  |  |  | Selected for Study | Failed in Genotyping | With non-Polymorphic Locus | Filtered out^*^ | Successfully Genotyped |  |
|  |  |  |  |  |  |  |  |  |  |
| AHCY | 20 | 23 | S-adenosyl-homocysteine hydrolase | 14 | 2 | 3 | 0 | 9 | 1 |
| AMD1 | 6 | 21 | S-adenosyl-methionine decarboxylase 1 | 17 | 2 | 3 | 2 | 10 | 4 |
| BHMT | 5 | 21 | betaine-homocysteine methyltransferase | 14 | 0 | 2 | 2 | 10 | 8 |
| CBS | 21 | 23 | cystathionine-beta-synthase | 14 | 1 | 0 | 3 | 10 | 7 |
| CHDH | 3 | 28.5 | choline dehydrogenase | 20 | 2 | 2 | 2 | 14 | 10 |
| CTH | 1 | 28.3 | cystathionase | 16 | 0 | 1 | 0 | 15 | 6 |
| DHFR | 5 | 29.1 | dihydrofolate reductase | 13 | 1 | 0 | 4 | 8 | 2 |
| FOLR1 | 11 | 6.8 | folate receptor 1 | 7 | 1 | 2 | 0 | 4 | 4 |
| GIF | 11 | 16.2 | gastric intrinsic factor | 9 | 1 | 3 | 0 | 5 | 3 |
| MAT1a | 10 | 17.9 | methionine adenosyltransferase I, alpha | 16 | 1 | 3 | 2 | 10 | 4 |
| MAT2a | 2 | 6.1 | methionine adenosyltransferase II, alpha | 9 | 0 | 1 | 1 | 7 | 2 |
| MAT2b | 5 | 16.1 | methionine adenosyltransferase II, beta | 11 | 3 | 3 | 0 | 5 | 4 |
| MTHFD1 | 14 | 71.7 | methylenetetra-hydrofolate dehydrogenase 1 | 31 | 1 | 3 | 1 | 26 | 15 |
| MTHFD2 | 2 | 16.7 | methylenetetra-hydrofolate dehydrogenase 2 | 9 | 0 | 4 | 0 | 5 | 5 |
| MTHFR | 1 | 20.3 | 5,10-methylene-tetrahydrofolate reductase | 16 | 0 | 2 | 2 | 12 | 5 |
| MTR | 1 | 109 | 5-methyltetra-hydrofolate-homocysteine methyltransferase | 49 | 2 | 7 | 0 | 40 | 12 |
| MTRR | 5 | 32 | 5-methyltetra-hydrofolate-homocysteine methyltransferase reductase | 19 | 1 | 2 | 0 | 16 | 9 |
| NNMT | 11 | 54.7 | nicotinamide N-methyltransferase | 35 | 13 | 9 | 1 | 12 | 8 |
| PON1 | 7 | 26.9 | paraoxonase 1 | 18 | 1 | 1 | 0 | 16 | 11 |
| SHMT1 | 17 | 35.7 | serine hydroxymethyl-transferase 1 | 18 | 0 | 4 | 3 | 11 | 5 |
| SHMT2 | 12 | 5.2 | serine hydroxymethyl-transferase 2 | 8 | 2 | 4 | 1 | 1 | 1 |
| SLC19A1 | 21 | 48.9 | solute carrier family 19 member 1 | 15 | 1 | 1 | 0 | 13 | 7 |
| TCN1 | 11 | 13.7 | transcobalamin I | 7 | 1 | 2 | 0 | 4 | 2 |
| TCN2 | 22 | 19.9 | transcobalamin II | 14 | 1 | 0 | 2 | 11 | 5 |
| TYMS | 18 | 15.9 | thymidylate synthase | 18 | 5 | 2 | 0 | 11 | 7 |
| **Total** |  |  |  | **417** | **42** | **64** | **26** | **285** | **147** |

^*^ SNPs with MAF<0.01 or HWE<1.6x10^-4^ were excluded from the analysis.
