# Supplementary material for: A Comprehensive Association Analysis of Homocysteine Metabolic Pathway Genes in Singaporean Chinese with Ischemic Stroke
Source: PLoS One. 2011 Sep 15;6(9):e24757. doi: 10.1371/journal.pone.0024757 (PMC3174208; doi:10.1371/journal.pone.0024757)
Supplement: Table S3 — SNPs selected for replication study. (DOCX) [file pone.0024757.s004.docx]

|  | | | |  |  |  |
| --- | --- | --- | --- | --- | --- | --- |
| SNPs ID | Gene | Remarks |  |  |  |  |
| rs16879259 | MTRR | tagging SNP of significant SNP rs16879248 (r2=1) |  |  |  |  |
| rs2301955 | TCN2 | tagging SNP of significant SNP rs11703570 (r2=1) |  |  |  |  |
| rs9909104 | SHMT1 | tagging SNP of significant SNP rs11868708 (r2=1) |  |  |  |  |
| rs5749131 | TCN2 | SNP in significant haplotype |  |  |  |  |
| rs502396 | TYMS | SNP in significant haplotype |  |  |  |  |
| rs2853532 | TYMS | SNP in significant haplotype |  |  |  |  |
| rs2273028 | SHMT1 | SNP in significant haplotype |  |  |  |  |
| rs2273026 | SHMT1 | SNP in significant haplotype |  |  |  |  |
