# Supplementary material for: A Comprehensive Association Analysis of Homocysteine Metabolic Pathway Genes in Singaporean Chinese with Ischemic Stroke
Source: PLoS One. 2011 Sep 15;6(9):e24757. doi: 10.1371/journal.pone.0024757 (PMC3174208; doi:10.1371/journal.pone.0024757)
Supplement: Table S4 — Association of 3 Significant SNPs with Ischemic Stroke Susceptibility under Different Inheritance Models. (DOCX) [file pone.0024757.s005.docx]

|  | | |  |  | **Additive Model** | |  | **Dominant Model** | |  | **Recessive Model** | |
| --- | --- | --- | --- | --- | --- | --- | --- | --- | --- | --- | --- | --- |
|  |  |  | MAF_control_ | MAF_case_ | P | OR^#^ |  | P | OR^#^ |  | P | OR^#^ |
| dbSNP ID | Gene | Alleles^a^ |  |  |  | (95% CI) |  |  | (95% CI) |  |  | (95% CI) |
| rs16879248 | *MTRR* | T/C | 0.22 | 0.18 | 0.004 | 0.75 |  | 0.003 | 0.71 |  | 0.254 | 0.80 |
|  |  |  |  |  |  | (0.61-0.91) |  |  | (0.56-0.89) |  |  | (0.43-1.49) |
| rs11868708 | *SHMT1* | T/C | 0.31 | 0.35 | 0.010 | 1.24 |  | 0.010 | 1.33 |  | 0.225 | 1.28 |
|  |  |  |  |  |  | (1.05-1.46) |  |  | (1.07-1.66) |  |  | (0.91-1.81) |
| rs11703570 | *TCN2* | T/A | 0.18 | 0.2 | 0.018 | 1.28 |  | 0.011 | 1.35 |  | 0.386 | 1.13 |
|  |  |  |  |  |  | (1.04-1.57) |  |  | (1.07-1.7) |  |  | (0.58-2.21) |

^#^ Adjusted for study and risk factors: gender, age, hypertension, diabetes, hyperlipidemia, smoking.
