# Supplementary material for: A Comprehensive Association Analysis of Homocysteine Metabolic Pathway Genes in Singaporean Chinese with Ischemic Stroke
Source: PLoS One. 2011 Sep 15;6(9):e24757. doi: 10.1371/journal.pone.0024757 (PMC3174208; doi:10.1371/journal.pone.0024757)
Supplement: Table S5 — Power Calculation for Two-stage Case-Control Association Study (Stage 1: 360 Cases & 354 Controls; Stage 2: 420 Cases & 420 Controls) at a Prevalence of 3% (Significant Level = 0.0005, 147 SNPs). (DOCX) [file pone.0024757.s006.docx]

| **OR** | **Power** | |
| --- | --- | --- |
|  | **MAF=0.1** | **MAF=0.2** |
| 1.2 | 3% | 9% |
| 1.3 | 13% | 34% |
| 1.4 | 33% | 69% |
| 1.5 | 59% | 90% |
