# Supplementary material for: A Comprehensive Association Analysis of Homocysteine Metabolic Pathway Genes in Singaporean Chinese with Ischemic Stroke
Source: PLoS One. 2011 Sep 15;6(9):e24757. doi: 10.1371/journal.pone.0024757 (PMC3174208; doi:10.1371/journal.pone.0024757)
Supplement: Text S1 — Supplementary information of method. (DOCX) [file pone.0024757.s007.docx]

**METHODS**

**DNA Extraction**

20 ml of fasting venous blood samples were collected in EDTA anti-coagulant tubes. These were immediately placed on ice and transported to the laboratory where they were subsequently centrifuged at 4000 rpm for 10 minutes at 4°C. Plasma and whole blood cells were separated and stored at -80°C until analysis. 4 ml of the latter was used in genomic DNA extraction where isolation was carried out using commercially available QIAamp DNA Blood Mini Kit (Qiagen, GmbH, Hilden, Germany) according to the manufacturer’s protocol. Isolated genomic DNA was then kept at 4°C.

**SNP Selection and Genotyping**

In the initial study, 417 SNPs from the 25 homocysteine metabolic genes (*Figure 1*, *Table S2*) were selected for genotyping analysis. Genotyping analyses were carried out in 746 samples of the initial study (384 cases and 362 controls) using the MassArray system from Sequenom (San Diego, USA) and the GoldenGate Assay from Illumina (San Diego, USA) according to the manufacturer’s instructions. DNA samples were randomly assigned to genotyping DNA plates each with both positive and negative controls. All the genotyping results were generated and checked by laboratory staff blinded to the case-control status. 32 samples with a SNP call rate of less than 90% were eliminated from further analysis. Similarly, 42 SNPs with a genotype call rate less than 90% were eliminated from further statistical analysis. In addition, there were 64 SNPs found to be non-polymorphic in our samples. This high frequency of non-polymorphic SNPs is attributable to selection of SNPs from the dbSNP database which had a high proportion of unverified SNP information. Using a minor allele frequency (MAF) of 0.01 and a Hardy-Weinberg Equilibrium (HWE) p-value of 1.6x10^-4^ (0.05/311, in controls) as cutoff values, a further 26 SNPs were excluded. Hence, in total, 132 SNPs were removed from further analysis (*Table S2)*. In the initial study, the final dataset for association analysis contains the genotyping results of 285 SNPs in 714 samples.

In the replication study, 8 SNPs were selected and analyzed to validate the associations observed in the initial study (*Table S3*). All the controls of the replication study had already been genotyped using Illumina Human1M-Duo DNA Analysis BeadChip. The 3 significant SNPs (rs16879259, rs2301955, rs9909104) from the initial study were, however, not in the Human1M-Duo BeadChip, therefore, we selected tagging SNPs of the Human1M-Duo BeadChip which have pairwise r^2^=1 with them (calculated from HapMap CHB data). 2 SNPs from *MTRR* haplotype (rs16879258, rs3822444) were not analyzed in the replication study, because none of the SNPs present in the Human1M-Duo BeadChip could tag them effectively (r^2^=0.8). Genotyping analysis of the 8 SNPs were carried out in the stroke samples of the validation study using the MassArray system from Sequenom (San Diego, USA). All SNPs have genotyping call rate > 98%, MAF>0.01 and HWE > 0.005.

**Principle Component Analysis**

We performed PCA (Principal component analysis)^1^ in the initial samples using genotype data of 147 tagging SNPs. All the 714 samples (369 cases and 354 controls) were analyzed together with 194 reference samples (of European, Chinese, Japanese and Yoruban ancestries) from the International HapMap Project (*Figure S1*).

**REFERENCE**

1. Price, A.L. et al. Principal components analysis corrects for stratification in genome-wide association studies. *Nat Genet* **38**, 904-9 (2006).
